# Heparanase induces necroptosis of microvascular endothelial cells to promote the metastasis of hepatocellular carcinoma

**DOI:** 10.1038/s41420-021-00411-5

**Published:** 2021-02-17

**Authors:** Xiaopeng Chen, Bin Cheng, Dafei Dai, Yuhai Wu, Zhiwen Feng, Chaogang Tong, Xiangming Wang, Jun Zhao

**Affiliations:** 1 First Department of Hepatobiliary Surgery, Affiliated Yijishan Hospital of Wannan Medical College, 241001 Wuhu, China; 2 Department of Hepatobiliary surgery, Huangshan People’s Hospital, 245000 Huangshan, China; 3 Department of General Surgery, Affiliated Chaohu Hospital, Anhui Medical University, 238000 Hefei, China; 4 Department of Pathology, Affiliated Yijishan Hospital of Wannan Medical College, 241001 Wuhu, China; 5 Department of Gastrointestinal Surgery, Affiliated Yijishan Hospital of Wannan Medical College, 241001 Wuhu, China

**Keywords:** Cancer microenvironment, RNAi

## Abstract

Heparanase (HPSE) is a kind of multifunctional extracellular hydrolase, and related to metastasis of hepatocellular carcinoma (HCC). Endothelial necroptosis promotes the metastasis of cancer cells. It is not clear whether HPSE could mediate necroptosis of microvascular endothelial cells (MVECs) to promote HCC metastasis. Here we found HPSE expression was up-regulated in HCC tissues and its over-expression was correlated with multiple tumor foci, microvascular invasion, and poor outcome of HCC patients. Non-contact co-culture experiments showed high-expressed HPSE in HCC cells mediated the necroptosis of human umbilical vein endothelial cells (HUVECs) and elevated the expression levels of syndecan-1 (SDC-1) and tumor necrosis factor-α (TNF-α) in vitro. As a result of necroptosis, trans-endothelial migration (TEM) of HCC cells was increased. Conversely, both HPSE and SDC-1 knockdowns reversed necroptosis and decreased TNF-α expression level, while HPSE over-expression increased SDC-1 and TNF-α expression and aggravated necroptosis. Animal experiments found that the nude mice, intraperitoneally injected with HPSE high expressing HCC cells, had obvious necroptosis of MVECs and high intrahepatic metastasis rate, which could be relieved by inhibitor of necroptosis. Morever, HPSE elevated the expression levels of p38 mitogen-activated protein kinase (p38 MAPK) rather than nuclear factor kappa B in vitro. Our data suggest that HPSE induces necroptosis of MVECs to promote the metastasis of HCC by activating HPSE/SDC-1/TNF-α axis and p38 MAPK pathway.

## Introduction

Hepatocellular carcinoma (HCC) is a kind of high malignant tumors with an extremely poor prognosis^1,2^, while intrahepatic metastasis or recurrence is the main cause to be blamed for this situation. HCC metastasis is a complex multistep process, and the physical displacement of HCC cells from portal vein microcirculation to new liver tissue across the endothelial barrier is one of the key steps^3^.

Heparanase (HPSE) is a kind of multifunctional extracellular hydrolase, which is up-regulated in almost all human malignant tumors^4^. As the hydrolytic substrate of HPSE, heparan sulfate proteoglycans (HSPGs), composed by a core protein with covalently attached heparan sulfate (HS) side chains, are widely distributed in the extracellular matrix (ECM) and cell surface^5^. The HS side chains are able to bind to a variety of proteins, including basic fibroblast growth factor (bFGF), vascular endothelial growth factor (VEGF), and tumor necrosis factor-α (TNF-α), thus providing a local extracellular storage depot in various tissues^5^. Based on the property of HS side chain, HPSE promotes tumor growth, metastasis, and angiogenesis by degrading HSPGs and releasing VEGF and bFGF from the extracellular cytokines depot^6^.

Our previous studies demonstrated that HPSE was closely related to metastasis and prognosis of HCC patients^7^. Further research found that HPSE could induce the formation of portal venous microemboli and trans-endothelial migration (TEM) of HCC cells^3^. However, the exact mechanisms remain undetermined. After entering microcirculation, the metastatic potential of HCC cells mainly depends on a rapid and efficient way to escape from the microvessel by crossing the endothelial barrier^8,9^. It is reported that endothelial permeability elevation achieved by opening or expanding of intercellular space via various mechanisms was beneficial for cancer cells to extravasate^10^. Recently, it is found that cancer cell induces endothelial cell necroptosis which promotes TEM and metastasis of cancer cells^11^. TNF-α is the most common initiator of necroptosis. Studies confirm that HPSE promotes the expression and secretion of TNF-α, and HPSE knockdown inhibits TNF-α expression^12^. Syndecan-1 (SDC-1) is one of the important substrates of HPSE on the cell surface, and its HS chains also can bind to growth factors, chemokines, and cytokines^13^. When the HS side chains of SDC-1 are degraded by HPSE, its binding cytokines such as TNF-α are released. Moreover, the recent bioinformatic analysis demonstrates that SDC-1 can interact with TNF family members^14^, and induce the effective biological effect. On the other hand, p38 mitogen-activated protein kinase (p38 MAPK) is an important cell signaling pathway related to necroptosis^15^, and closely associated with liver metastasis^16^. However, the relationship between these molecules and necroptosis of endothelial cell in HCC has not yet been studied.

In the current study, we found that high-expressed HPSE in HCC cells may induce necroptosis of the adjacent microvascular endothelial cells (MVECs) to promote intrahepatic metastasis of HCC by activating the HPSE/SDC-1/TNF-α axis and p38 MAPK pathway. Our findings provide new insight into HCC metastasis and suggest candidate target for potential use in HCC therapy in the future.

## Results

### HPSE expression is up-regulated in HCC

**Fig. 1:**
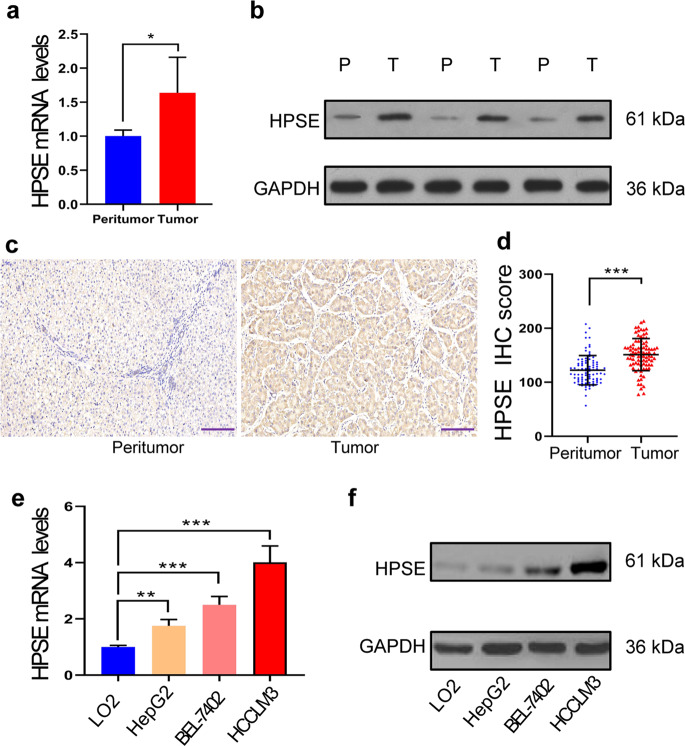
HPSE expression is up-regulated in HCC. **a**, **b** HPSE mRNA (**a**) and protein (**b**) expression levels in peritumor tissues (P) and tumor tissues (T) of 6 HCC patients were detected by qRT-PCR and western blotting, respectively. **c** HPSE expressions in peritumor tissues and tumor tissues of 88 HCC patients were measured by IHC assay (scale bars, 200 μm). **d** The average IHC score of HCC tissues of 88 patients was significantly higher than that of peritumor tissues. **e**, **f** HPSE mRNA (**e**) and protein (**f**) expression levels in LO2 cell line and three kinds of HCC cell lines were measured by qRT-PCR and western blotting, respectively. *P < 0.05, **P < 0.01, and ***P < 0.001.

In this study, we first examined the mRNA and protein expression levels of HPSE in six pairs of human HCC tissues and matched peritumor tissues. The results revealed that HPSE expression levels were markedly up-regulated in HCC tissues (Fig. 1a, b). Then, we further detected HPSE protein expression levels in the tumors of 88 HCC patients using IHC staining, and confirmed that the average HPSE IHC score in tumor tissues was significantly higher than that in peritumor tissues (Fig. 1c, d). HPSE mRNA and protein expression levels were also increased in all three HCC cell lines compared with those in normal human liver cell lines. The highest HPSE expression was found in the HCCLM3 cell line while the lowest was in the HepG2 cell line (Fig. 1e, f).

### HPSE promotes invasive metastasis of HCC cells

**Fig. 2:**
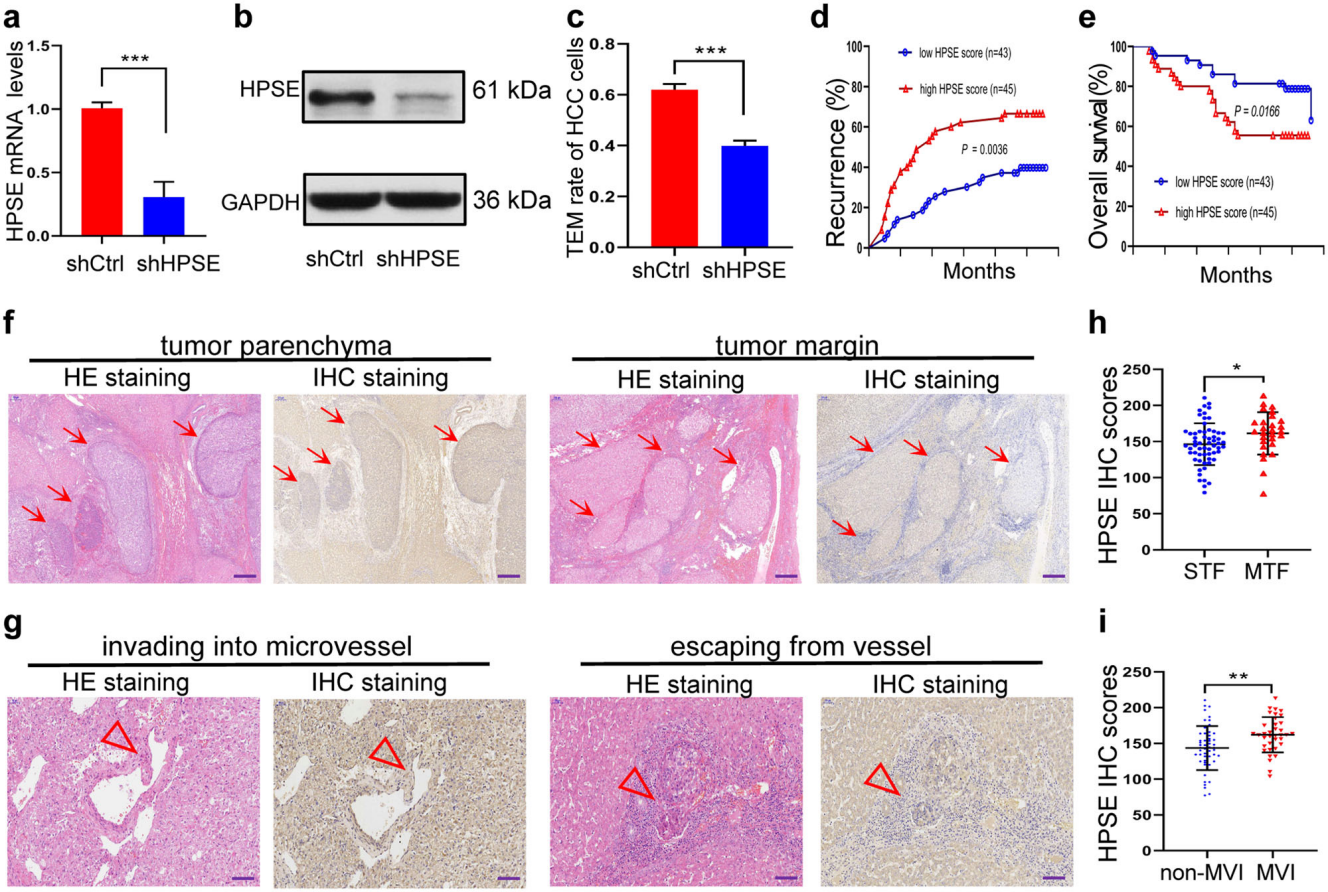
HPSE expression is correlated with invasive metastasis. **a**, **b** HPSE mRNA (**a**) and protein (**b**) levels in lentivirus shRNA transfected HCC cells were detected by qRT-PCR and western blotting, respectively. **c** TEM rate of transfected HCC cells was measured by CCK-8 assay. **d**, **e** Recurrence (**d**) and survival probability (**e**) of HCC patients. **f**, **g** Multiple tumor foci (MTF) (**f**, red arrow, scale bar: 400 μm) and MVI (**g**, red triangle, scale bar: 100μm) were detected by HE and IHC staining of HCC tissues. **h**, **i** IHC score in MTF (**h**)/MVI group (**i**) was obviously higher than that in STF/non-MVI group. *P < 0.05, **P < 0.01, and ***P < 0.001.

To prove whether HPSE promotes invasive metastasis of HCC cells, HPSE high expressing HCCLM3 cell line was first transfected with HPSE shRNA vector. After transfection, the HPSE mRNA and protein expression levels were markedly decreased (Fig. 2a, b). It was found in the following TEM experiment that the TEM rate of HCC cells in the shHPSE group was significantly lower than that in the shCtrl group (Fig. 2c). These results suggest that HPSE promotes the invasion and migration of HCC cells.

To further prove whether HPSE promotes HCC metastasis, above-mentioned data from 88 patients were then divided into low HPSE expression group (*n* = 43) and high expression group (*n* = 45) according to the average value (151.15) of IHC score. Correlation between the clinicopathologic characteristics of 88 HCC patients and HPSE expression was shown in Supplementary Table S1. HPSE expression was closely correlated with Edmondson’s classification (*P* = 0.0318), AJCC staging of HCC (8th edition) (*P* = 0.0375), multiple tumor foci (MTF) (*P* = 0.0093), and microvascular invasion (MVI) (*P* = 0.0043). Patients with high HPSE expression had a higher recurrence probability and poorer overall survival (OS) than those with low HPSE expression (Fig. 2d, e). MTF (Fig. 2f) and MVI including tumor thrombus (Fig. 2g) were common signs in the high HPSE expression group. After further dividing patients into two subgroups based on multiple or single tumor foci (STF), we found that the MTF group had a higher expression level of HPSE than that in the STF group (Fig. 2h). Compared with the non-MVI group, HPSE level in the MVI group was markedly increased (Fig. 2i). These data indicate that HPSE induces the necrosis or damage of MEVCs to promote the invasion and metastasis of HCC cells.

### HPSE induces necroptosis of HUVECs in vitro

**Fig. 3:**
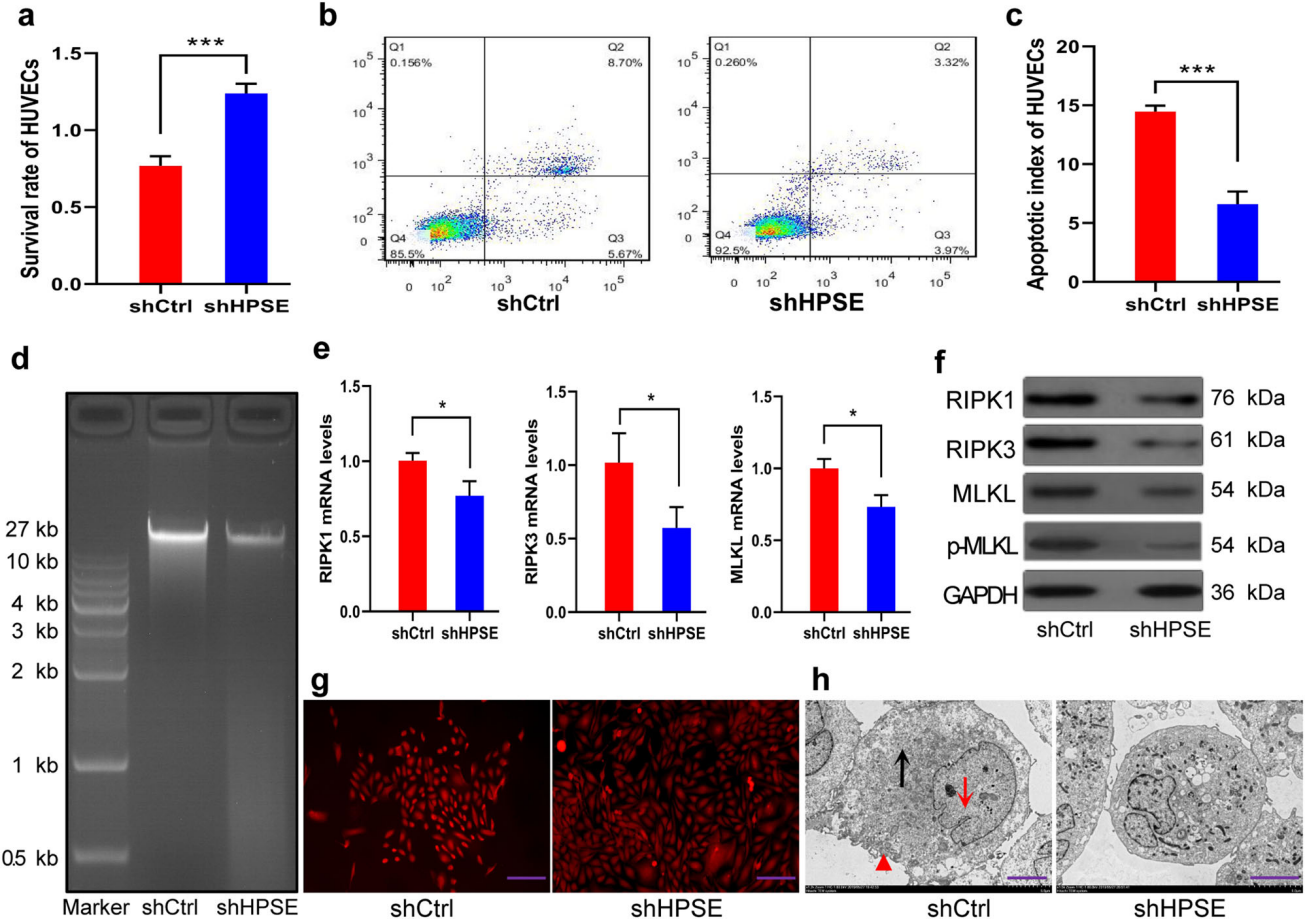
HPSE induces necroptosis of HUVECs in vitro. **a** The survival rate of HUVECs was determined by CCK-8 assay. **b** Flow cytometry dot plot of HUVECs in two groups. **c** Apoptotic index of HUVECs in shCtrl group was significantly higher than that in shHPSE group. **d** DNA electrophoretogram of HUVECs of both groups showed only a long DNA fragment (length 27 kb). **e**, **f** RIP1, RIP3, and MLKL (p-MLKL) mRNA (**e**) and protein (**f**) levels in HUVECs of two groups were detected by qRT-PCR and Western blotting, respectively. **g**, **h** Morphology of HUVECs were observed by fluorescence microscopy (**g**, scale bars: 50 μm) and transmission electron microscopy (**h**, scale bars: 5 μm), respectively. Representative images are shown. Membrane rupture (red triangle), release of the intracellular contents, cell swelling (black arrow), and nuclear fragmentation (red arrow) could be found in shCtrl group. *P < 0.05, **P < 0.01, and ***P < 0.001.

To investigate whether HPSE induces necroptosis of MEVCs, we used transfected HCCLM3 cells to co-culture in non-contact with HUVECs in a transwell chamber. CCK-8 assay showed that the survival rate of HUVECs in the shCtrl group was significantly lower than that in the shHPSE group (Fig. 3a), while the apoptotic index in the shCtrl group was markedly higher than that in the shHPSE group (Fig. 3b, c). DNA electrophoresis assay of HUVECs only showed a long DNA fragment (length >10 kb), and no degradation of genomic DNA was observed in both groups (Fig. 3d). These results suggested that HPSE mainly promoted the necrosis of HUVECs rather than apoptosis. Then, we further detected the RIPK1, RIPK3, and MLKL levels in HUVECs using qRT-PCR and western blotting. The results showed their mRNA and protein levels in the shCtrl group were markedly higher than those in the shHPSE group (Fig. 3e, f). With the decrease of MLKL expression level in the shHPSE group, pMLKL protein level also declined significantly (Fig. 3f). Under a fluorescence microscope, some HUVECs in the shCtrl group were damaged, and cytoplasm effused resulting in a decrease in the number of cells and enlargement of intercellular gaps. However, no obvious cell morphological abnormality was observed in the shHPSE group (Fig. 3g). Under transmission electron microscopy, the rupture of cytomembrane, swelling of cytoplasm, dissolving and condensing of mitochondria and Golgi apparatus, disappearance of cell structure and broken down of nucleus were found in the shCtrl group. However, cell morphology was basically normal in the shHPSE group (Fig. 3h). In summary, these results further indicate that HPSE induces the necroptosis of HUVECs in vitro, which could be alleviated by HPSE knockdown.

### HPSE mediates necroptosis of MVECs in vivo

**Fig. 4:**
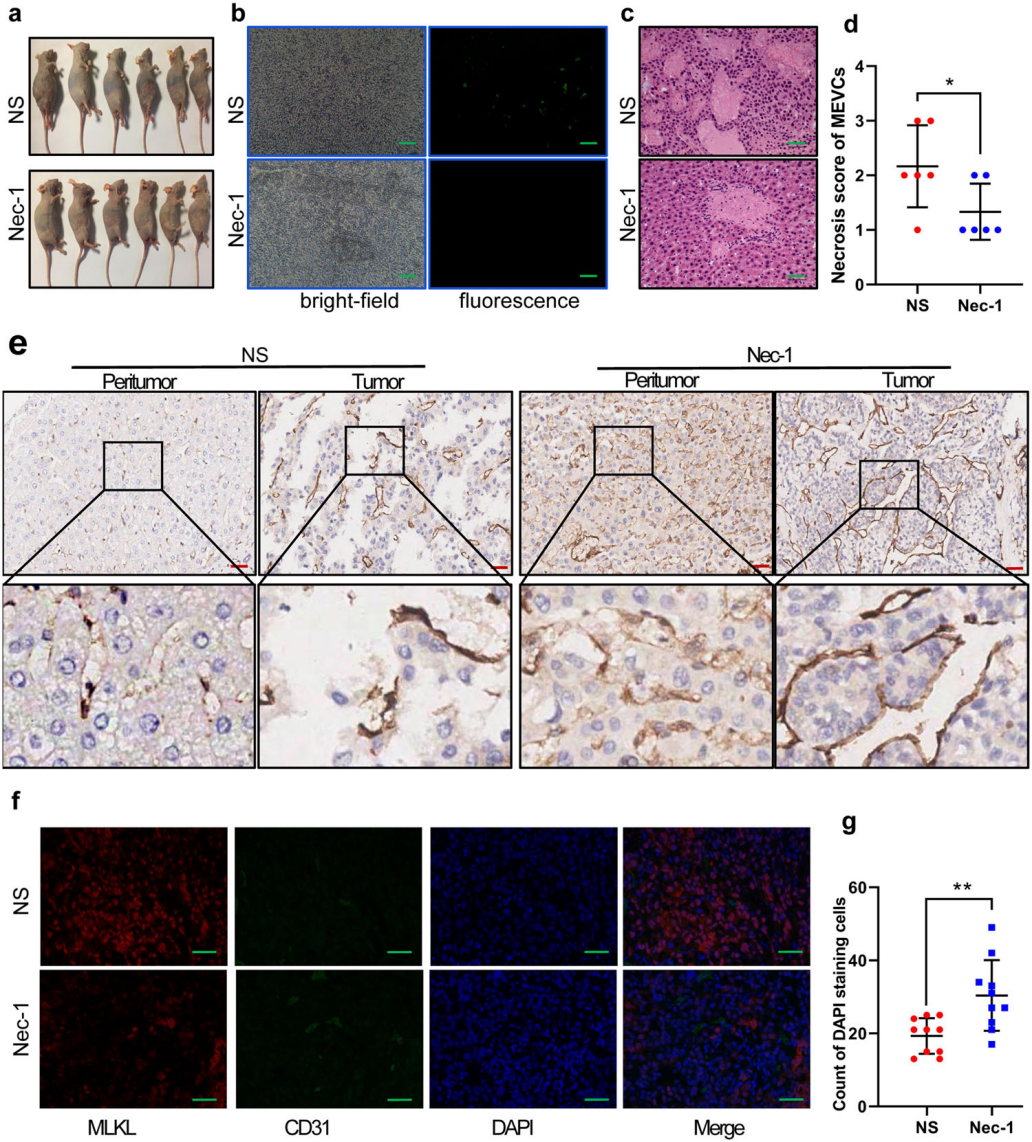
HPSE on necroptosis of vascular endothelial cells in vivo. **a** The two groups of nude mice showed ascites. **b** Histofluorescence showed GFP-positive HCC cells in the liver tissue of mice in NS group, and no positive cells in Nec-1 group (scale bars, 50 μm). **c** Representative images of HE staining of the liver tissues are shown (scale bars: 50 μm). **d** Necrosis score of MEVCs in NS group was significantly higher than that of Nec-1 group. **e** CD31 IHC staining showed that the integrity of liver microvessel in NS group was seriously damaged (scale bars: 200 μm). **f** Double immunofluorescent analysis showed the damage of microvessel integrity (CD31 staining, green) and increased necroptosis (MLKL staining, red) in NS group (scale bars: 50 μm). **g** Ten size-fixed fields (0.1 × 0.1 mm^2^) were randomly selected to quantify the number of DAPI-stained cells. *P < 0.05, **P < 0.01, and ***P < 0.001.

To further verify our findings, we performed animal experiments. All mice developed with cancerous ascites (Fig. 4a) and omental metastasis (Supplementary Fig. S1a,b) six weeks later after HCC cell inoculation. The tumor formation rates of abdominal cavity were both 100% in two groups (6/6 vs. 6/6). Cancerous nodule rate of liver surface in the NS group was significantly higher than that in the Nec-1 group (6/6 vs. 1/6) (*P* < 0.01). Fluorescence microscopy detected the GFP fluorescence positive rate of liver tissues in the NS group (6/6) was also higher than that in the Nec-1 group (2/6) (*P* < 0.05) (Fig. 4b). The results suggest that HPSE promotes tumor growth and liver metastasis of HCC cells, which could be effectively suppressed by Nec-1. Under the light microscope, HCC cells could be discovered in all liver tissues of the NS group (Supplementary Fig. S1c), and some MVECs showed necrosis, including cell swelling, disintegration, exfoliation, disappearance of cell structure and destruction of endothelial cell integrity (Fig. 4c). However, endothelial cell morphology in Nec-1 group turned to normal. The necrosis score of MEVCs in NS group was significantly higher than that of the Nec-1 group (Fig. 4d). CD31 IHC staining showed that the integrities of microvessel of peritumor and tumor tissues in the NS group were seriously damaged, while the microvessels in the Nec-1 group were basically intact (Fig. 4e). Moreover, double immunofluorescent analysis found that increased MLKL-labeled necrotic cells (red) were accompanied by obvious destruction of the integrity of CD31 labeled microvessel (green) in the NS group. However, endothelial cells in the Nec-1 group remained intact without obvious cell necrosis (Fig. 4f). Consistent with CD31 labeled cells, the number of DAPI-stained cells in the NS group was significantly less than that of the Nec-1 group (Fig. 4g). Collectively, HCCLM3 cells with high HPSE expression mediate necroptosis of MVECs to promote intrahepatic metastasis in vivo, which could be relieved by inhibitor of necroptosis.

### HPSE/SDC-1/TNF-α axis plays a crucial role in necroptosis of HUVECs in vitro

**Fig. 5:**
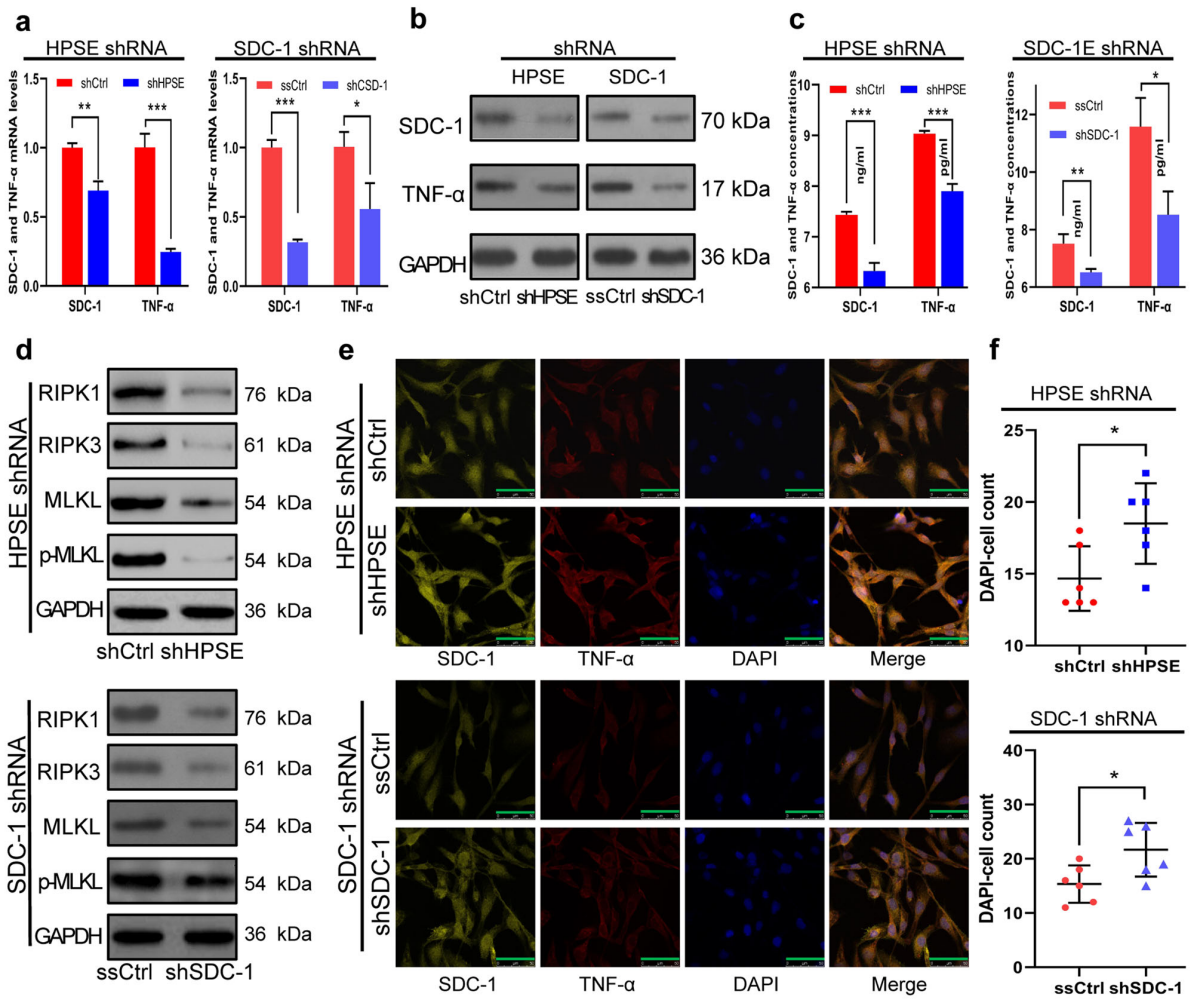
HPSE/SDC-1/TNF-α axis plays a crucial role in necroptosis of HUVECs in vitro. **a**, **b** SDC-1, TNF-α mRNA (**a**) and protein (**b**) expressions in HUVECs were detected by qRT-PCR and western blotting, respectively. **c** Soluble SDC-1 and TNF-α concentrations in the supernatant were detected by ELISA assay. **d** RIP1, RIP3, MLKL, and p-MLKL protein levels in HUVECs were determined by western blotting. **e** SDC-1 and TNF-α distributions in HUVECs were observed by fluorescence microscope (scale bars: 50 μm). **f** The number of DAPI staining HUVECs significantly increased after HPSE or SDC-1 expression was inhibited. *P < 0.05, **P < 0.01, and ***P < 0.001.

To uncover the activation mechanism underlying HPSE mediated necroptosis, we observed the changes of some downstream molecules of HPSE in HUVECs. The qRT-PCR showed that the SDC-1 mRNA level in co-cultured HUVECs of the shCtrl group was significantly higher than that in the shHPSE group (Fig. 5a), and western blotting demonstrated that SDC-1 protein level had a similar trend of change (Fig. 5b). In addition, soluble SDC-1concentration in the supernatant of the shCtrl group was also significantly higher than that in the shHPSE group (Fig. 5c). With the decreases of HPSE and SDC-1 levels in the shHPSE group, TNF-α mRNA and protein levels also declined (Fig. 5a, b), and TNF-α concentration in the supernatant showed the same change (Fig. 5c). As characteristic proteins of necroptosis, RIPK1, RIPK3, MLKL, and p-MLKL expressions in HUVECs showed similar changes (Fig. 5d). Double immunofluorescent analysis found that both SDC-1 and TNF-α were widely distributed in the cell membrane and cytoplasm of HUVECs (Fig. 5e). With the knockdown of HPSE in co-cultured HCC cells, the number of DAPI-positive cells significantly increased (Fig. 5f). These results suggest that HPSE induces necroptosis of HUVECs by elevating SDC-1 and TNF-α levels.

SDC-1 is one of the major HSPGs on the cell surface. To confirm the key role of SDC-1 in necroptosis, SDC-1 shRNA vector was transfected into HUVECs. The results found that the shRNA vector could effectively decrease the SDC-1 expression (Fig. 5a–c). With the knockdown of SDC-1, the TNF-α also showed corresponding changes (Fig. 5a–c). When SDC-1 shRNA vector transfected HUVECs was co-cultured with HCCLM3 cells, the RIPK1, RIPK3, MLKL, and p-MLKL expressions in HUVECs also decreased (Fig. 5d). Double immunofluorescent analysis showed similar phenomena as HPSE was knocked down in HCC cells (Fig. 5e), and the number of DAPI-stained cells significantly increased after SDC-1 expression was inhibited (Fig. 5f). These results suggest HPSE can not induce necroptosis of HUVECs if endothelial cell SDC-1 expression is inhibited in advance. Collectively, HPSE/SDC-1/TNF-α axis plays a crucial role in initiating necroptosis.

### Effect of HPSE over-expression on necroptosis of HUVECs in vitro

**Fig. 6:**
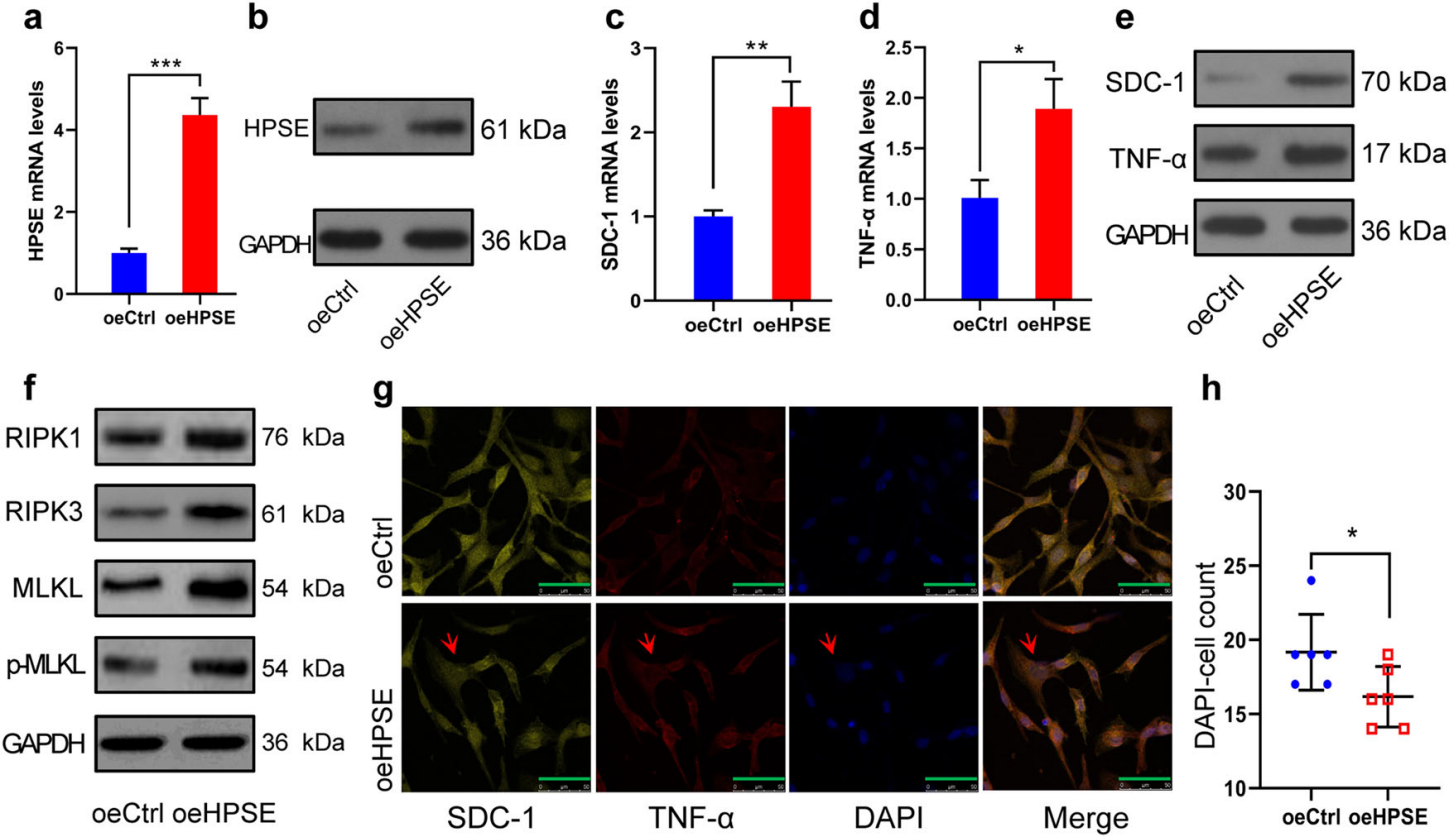
Effect of HPSE over-expression on necroptosis of HUVECs in vitro. **a**, **b** HPSE mRNA (**a**) and protein (**b**) expression levels in transfected HepG2 cells were detected by qRT-PCR and western blotting, respectively. **c**, **d** SDC-1 (**c**) and TNF-α (**d**) mRNA expression levels in HUVECs were detected by qRT-PCR after co-cultured with transfected HepG2 cells. **e**, **f** SDC-1, TNF-α (**e**), RIP1,RIP3, MLKL, and p-MLKL (**f**) protein levels in HUVECs were validated by western blotting. **g** SDC-1 and TNF-α distributions in HUVECs were observed by fluorescence microscope, extremely swollen nuclei (red arrow) was found in oeHPSE group (scale bars, 50 μm). **h** The number of DAPI staining HUVECs in oeHPSE group was significantly decreased compared with that in oeCtrl group. *P < 0.05, **P < 0.01, and ***P < 0.001.

To further confirm that HPSE mediates the necroptosis of HUVECs through HPSE/SDC-1/TNF-α axis, we performed a verifiable experiment by up-regulating HPSE expression level in HPSE low expressing HepG2 cells. The HepG2 cells were transfected with empty control and HPSE over-expression vector, respectively. The qRT-PCR and western blotting found that the over-expression vector effectively increased the HPSE mRNA and protein expression level (Fig. 6a, b). After HUVECs were co-cultured in non-contact with these HepG2 cells, both the SDC-1 and TNF-α mRNA expression levels in HUVECs showed obvious elevation (Fig. 6c, d), and their protein expressions showed similar changes (Fig. 6e). RIPK1, RIPK3, MLKL, and p-MLKL protein expressions in HUVECs were also increased when HPSE was up-regulated (Fig. 6f). Following double immunofluorescent analysis found extremely swollen nuclei in the oeHPSE group (Fig. 6g), and the number of DAPI-stained cells significantly decreased in the oeHPSE group (Fig. 6h). These results indicate that low-level necroptosis can be recovered by HPSE over-expression in HCC cells via the HPSE/SDC-1/TNF-α axis.

### HPSE affects the expressions of crucial proteins of necroptosis

**Fig. 7:**
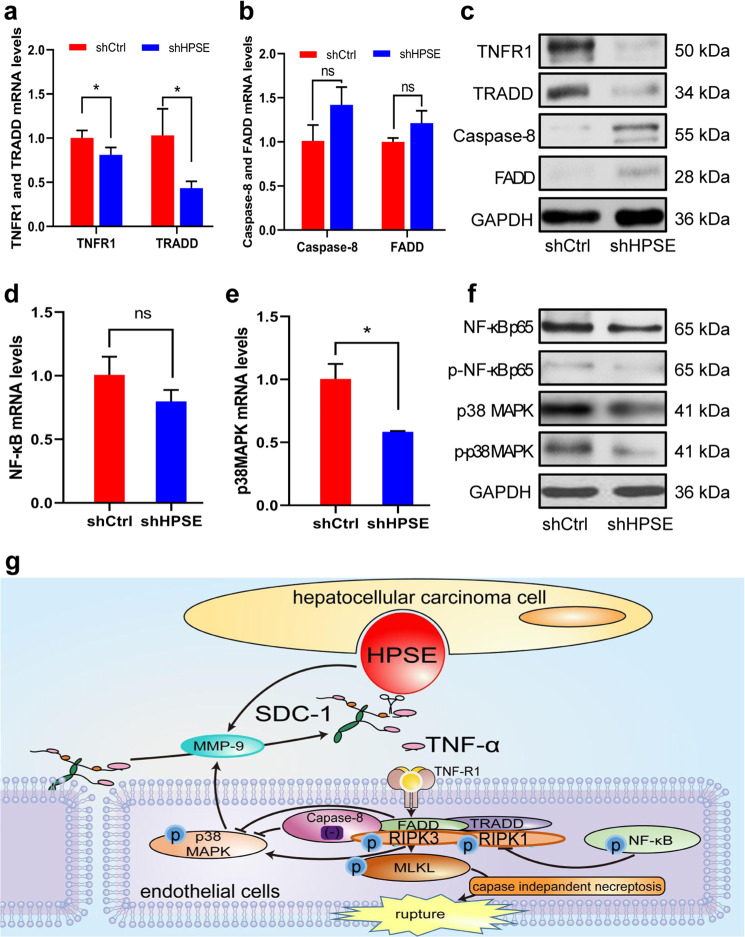
Expression of key proteins of necroptosis in HUVECs. **a**–**c** TNFR, TRADD (**a**), caspase-8 and FADD (**b**) mRNA and their protein expressions (**c**) in co-cultured HUVECs were validated by qRT-PCR and western blotting analysis, respectively. **d**–**f** NF-κB (**d**) and p38 MAPK mRNA (**e**) and their protein expressions (**f**) in co-culured HUVECs were detected by qRT-PCR and western blotting analysis, respectively. **g** The proposed model of HPSE/SDC-1/TNF-α axis and p38 MAPK pathway in HPSE induced necroptosis. *P < 0.05, **P < 0.01, and ***P < 0.001.

To understand the signal pathway of HPSE induced necroptosis in endothelial cells, we detected the mRNA and protein levels of some signaling proteins in HUVECs co-culured with transfected HCCLM3 cells. The qRT-PCR results found that the TNFR1 and TRADD mRNA levels in the shCtrl group were significantly higher than those in the shHPSE group (Fig. 7a), and the western blotting result showed similar changes (Fig. 7c). However, caspase-8 and FADD mRNA levels had no obvious difference between two groups (Fig. 7b), and both proteins were at lower levels (Fig. 7c). These results suggest that HPSE increases the expressions of TNFR1 and TRADD, but does not affect the activation of caspase-8 and FADD. Similar to caspase-8 and FADD expression, NF-κB and p-NF-κB expression had no obvious changes when HPSE expression was down-regulated (Fig. 7d, f). However, both the p38 MAPK mRNA and p-p38 MAPK protein levels showed a significant downregulation when HPSE expression was inhibited (Fig. 7e, f). These results indicate that p38 MAPK, but not NF-κB signal pathway, is activated in HPSE induced necroptosis (Fig. 7g).

## Discussion

HPSE is closely related to metastasis and prognosis of HCC, and high-level of HPSE predicts metastatic recurrence of HCC^4,6,7^. However, the underlying mechanisms still remain unclear. Strilic et al. recently reported that melanoma and lung cancer cells can mediate necroptosis of MEVCs resulting in extravasation and metastasis of cancer cells^9,11^. Necrosis or damage of MEVCs will enlarge the intercellular space, and the expanded space is more favorable for cancer cells to pass through the microvascular barrier and develop into metastasis sites. The results of the current study reveal that high-expressed HPSE in HCC cells can mediate necroptosis of MEVCs to promote the intrahepatic metastasis and postoperative recurrence of HCC.

In this study, we first demonstrated that HPSE was up-regulated in HCC tissues and cell lines. TEM test proved that the migration rate of HPSE high expressing HCC cells increased significantly. When HPSE expression was down-regulated, the TEM rate also decreased. Further clinical analysis showed that increased HPSE expression was closely correlated with MVI, intrahepatic MTF and postoperative recurrence of HCC patients. MVI and intrahepatic MTF were more common in the cancer tissues of HCC patients with high HPSE expression. The results indicate HPSE may mediate the necrosis or damage of MEVCs to promote the invasion and metastasis of HCC cells.

Then we performed in vitro and in vivo experiments to investigate whether HPSE could induce necroptosis of MEVCs of HCC. The survival analysis showed that high-expressed HPSE could significantly decrease the survival rate and increase the apoptotic index of co-cultured HUVECs. DNA electrophoresis assay did not find degradation of genomic DNA. These results suggest that HPSE mainly promotes the death rather than apoptosis of HUVECs. RIPK1 and RIPK3 are the vital proteins of necroptosis, which were related to the formation of “necrotic complex”, release of mitochondrial reactive oxygen species (ROS) and cell necrosis^17^. As the downstream executive protein of necroptosis, MLKL can ultimately lead to cell death and tissue damage after activated by RIPK3, and p-MLKL was one of the important markers of necroptosis^18^. Our following study showed that HPSE could elevate the RIPK1, RIPK3, MLKL (p-MLKL) mRNA and protein levels in HUVECs, suggesting that HPSE induces necroptosis of HUVECs. Necroptosis has the same morphological characteristics as necrosis^19^. Our morphological observation found HUVECs in the shCtrl group had typical cellular and subcellular morphological characteristics of necrosis. However, the cell morphology basically returned to normal when HPSE was down-regulated. The above results were well verified by the HPSE over-expression test using HepG2 cells. The findings indicate that HPSE can induce necroptosis of HUVECs in vitro. Furthermore, animal experiments proved that HPSE high expressing HCC cells could migrate from microcirculation of portal vein into liver parenchyma, accompanied by an increase of MLKL-labeled MEVCs and loss of endothelial integrity. The increase in the number of MLKL-labeled cells means aggravation of necroptosis. As an inhibitor of RIPK1, Nec-1 could specifically inhibit necroptosis^19^. In our experiments, the liver metastasis rate decreased significantly when necroptosis was inhibited in the Nec-1 group. Therefore, we think that HPSE can also mediate necroptosis of MEVCs in vivo.

In order to prove the role of the HPSE/SDC-1/TNF-α axis in HPSE induced necroptosis, we detected their expression levels in HCC cells and/or HUVECs, respectively. We found that TNF-α expression levels in HUVECs and supernatant were decreased after HPSE knockdown. The results demonstrate that HPSE increase the expression of TNF-α^12^. Metalloproteinase-9 (MMP-9) mediates the shedding of SDC-1 ectodomain into microenvironment, and HPSE increases the shedding of SDC-1 by pruning its HS chains^13^. In our study, we also found the soluble SDC-1 concentration in supernatant was obviously elevated in the shCtrl group. Surprisingly, the SDC-1 expression level in HUVECs of the shCtrl group was also obviously higher than that in the shHPSE group. These results confirm that HPSE not only increases the shedding of SDC-1 but also influenced its expression in HUVECs, which were consistent with the findings of Mahtouk K^13^. Furthermore, TNF-α expression level in HUVECs was also decreased after SDC-1 was knocked down. The following research confirmed RIPK1, RIPK3, MLKL, and p-MLKL levels significantly decreased after HCC cell HPSE or endothelial cell SDC-1 was knocked down in vitro. Immunofluorescent analysis found that both SDC-1 and TNF-α were widely distributed in the cell membrane and cytoplasm of HUVECs. With the knockdown of HPSE or SDC-1, the number of DAPI-stained endothelial cells significantly increased. Similar results were obtained by over-expression test using HepG2 cells. Based on these findings, we conclude HPSE/SDC-1/TNF-α axis plays a crucial role in the activation of necroptosis.

Necroptosis is a type of caspase-independent programmed cell deaths, and can be induced by many factors including specific death receptor and its ligand^19^. TNF-α/TNFR has been attracted much attention recently. Studies show that TNF-α binds to extracellular TNFR1, which leads to TNFR1 trimerization and the recruitment of intracellular TRADD to TNFR1. Our study showed that TNFR and TRADD expression levels were significantly up-regulated in HUVECs of shCtrl group, suggesting TNFR and TRADD play important roles in HPSE induced necroptosis. RIPK1, RIPK3 and are the most conversion molecules regulating apoptosis and necroptosis^17^. As a key factor of death receptor mediated extrinsic apoptosis pathway, FADD can recruit caspase-8, and FADD/caspase-8 can negatively regulate the expression of RIPKl and RIPK3^20^. Caspase-8 deficiency increases the sensitivity of intestinal epithelial cells to necroptosis^21^. In this study, we found both FADD and caspase-8 expressions in HUVECs were at lower levels and not affected by HPSE knockdown. Our data proved that low levels of FADD and caspase-8 were basic condition for HPSE induced necroptosis, which is consistent with the literature^22^. NF-κB pathway is related to cell survival. If RIPK1 is deubiquitinated by cylindromatosis, NF-κB mediated cell survival pathway is terminated^23^. In this study, we found the NF-κB expressions in HUVECs were not affected by HPSE knockdown, and p-NF-κB protein was at a low level. However, both the p38 MAPK and p-p38 MAPK levels in the shCtrl group were significantly up-regulated in our study. p38 MAPK is another important cell signaling pathway related to necroptosis1, and closely associated with liver metastasis^15,16^. These results indicate the NF-κB pathway is not activated and p38 MAPK pathway plays an important role in HPSE induced necroptosis (Fig. 7g).

In conclusion, HPSE mediates necroptosis of MEVCs to promote the intrahepatic metastasis and postoperative recurrence of HCC via activating the HPSE/SDC-1/TNF-α axis and p38 MAPK signaling pathway. To date, our finding of the new biological function of HPSE will help to enhance our understanding of HCC metastasis and likely provide a basis for developing anti-metastatic drugs for HCC.

## Materials and methods

### Patients and tissue selection

The clinical study was approved by the Ethics Committee of the Yijishan Hospital. Eighty-eight patients with HCC received curative operation at Yijishan Hospital, Wannan Medical College (Wuhu, China) between January 2015 and December 2016. Their tumor and peritumor tissues were collected for Hematoxylin-eosin (H&E) and immunohistochemistry (IHC) staining. Six pairs of fresh HCC tissues and peritumor tissues were randomly obtained from 88 patients for quantitative real-time PCR (qRT-PCR) and western blotting. The median follow-up time was 28 (3–67) months. The clinicopathological features of 88 patients are summarized in Supplementary Table S1.

### Cell culture

Human normal liver cell line LO2, human umbilical vein endothelial cells (HUVECs), HCC cell line BEL-7402, HCCLM3 and HepG2 were obtained from ATCC. The cells were cultured in DMEM/F12 + GlutaMax™ (027102, Gibco, CA, USA) supplemented with 10% FBS, 100 IU/ml penicillin and 100 mg/mL streptomycin at 37 °C in a humidified incubator with 5% CO_2_. When cells reached 70–80% confluence, they were harvested using 0.25% trypsin with 0.01% EDTA and seeded (1:2) into new culture flasks with complete DMEM. The media were replaced every 2 days.

### qRT-PCR

qRT-PCR was performed to detect the HPSE mRNA expression in liver tumor tissues, LO2 cell, BEL-7402, HCCLM3 and HepG2 cells. qRT-PCR was also used to determine the following gene mRNA expressions in HUVECs: SDC-1, TNF-α, receptor-interacting protein kinase 1(RIPK1), RIPK3, mixed lineage kinase domain-like protein (MLKL), TNF receptor 1 (TNFR1), TNFR associated death domain (TRADD), Caspase-8, Fas-associated death domain (FADD), p38 mitogen-activated protein kinase (p38 MAPK), and nuclear factor kappa B (NF-κB). Total RNA was extracted using TRIzol^®^ Plus RNA Purification Kit (12183-555, Invitrogen, Carlsbad, CA, USA). Reverse transcription was performed using the SuperScript III Reverse Transcriptase (11752-050, Invitrogen, Carlsbad, CA, USA) according to the instructions. Real-time PCR analysis was performed in triplicate using the SYBR® Green PCR Master Mix (4367659, ABI, Carlsbad, CA, USA) on an iCycler real-time Thermal Cycler (Bio-Rad Laboratories, Hercules, CA, USA). β-actin or glyceraldehyde-3-phosphate dehydrogenase (GAPDH) was used as an internal control. The primers were synthesized by Sangon Biotech Co., Ltd. (Shanghai, China) and described in Supplementary table S2.

### Western blotting analysis

Western blotting analysis was conducted as previously described^3^. Antibodies of HPSE (ab232817), GAPDH (ab181602), MLKL (ab184718), phosphorylated MLKL (p-MLKL) (ab196436), TNFR1(ab223352), TRADD (ab238960), caspase-8 (ab227430) and FADD (ab108601) were purchased from Abcam (Cambridge, MA, USA). SDC-1 (10593-1-AP), TNF-α (60291-1-AP) antibodies were from Proteintech Group, Inc (Rosemont, IL, USA). RIPK1 (A7414), RIPK3 (A12996), NF-κB (A19653), p-NF-κB(AP0124), p38 MAPK (A14401), and p-p38 MAPK (AP0526) antibodies were from ABclonal Technology (Woburn, MA, USA).

### Immunohistochemistry analysis

IHC analyses of HPSE and CD31 were performed by tissue microarray, and specific approaches were described in the literature^7^. A Aperio VERSA digital pathology scanner (Vista, CA, USA) was used to scan the entire slide and get digital files. The HPSE expression was assessed by histological score system^24^. The formula for the histological score is: IHC score = ∑(I × Pi), where I = intensity of staining and Pi = percentage of stained tumor cells, producing a cytoplasmic score ranging from 0 to 300. The scoring was independently assessed by two assessors who were not aware of the clinical outcomes.

### Lentivirus transfection assay

Human HPSE short hairpin RNA (shRNA) lentiviral vector, SDC-1 shRNA vector, recombinant HPSE over-expression vector and their control vectors were constructed and identified by Jikai Gene Biology Co., Ltd. (GXDL0145239, GXDL0174877 and GXDL0174876, Shanghai, China). They all contained a green fluorescent protein (GFP) coding sequence. The target sequences of HPSE and SDC-1 shRNA were 5′-TTTATGTGGCTGGATAAAT-3′ and 5′-GAGCAGGACTTCACCTTTGAA-3′, respectively. Their scramble sequences were both 5′-TTCTCCGAACGTGTCACGT-3′. Over-expression vector of HPSE contained a full-length cDNA of HPSE, and an empty vector was used as negative control. The 3 lentiviral vectors were transfected into HCCLM3 cells, HUVECs, and HepG2 cells, respectively. The transfection was performed according to the instructions. The transfection efficiency was monitored according to the expression of GFP by a fluorescence microscopy imaging system (Olympus Optical Co., Ltd., Tokyo, Japan).

### TEM assay

HUVEC-C cells (5×10^5^/well) were cultured in the upper chamber of 24-well transwell plate with 8 μm pore polycarbonate membrane insert. The cells were labeled with a Did fluorescent probe (AAT-22033, AAT Bioquest. Sunnyvale, CA, USA) according to the manufacturer’s instructions, and analyzed by fluorescence microscopy imaging system. The transfected HCCLM3 cells (1.2×10^5^/well) were then added to the upper chamber of transwell, and the DMEM medium containing 20% FBS was added to the lower chamber. After incubating for 24 h at 37 °C in a 5% CO_2_ incubator, the medium was abandoned. The lower chamber was added with 400 μL10% Cell Counting Kit-8 (CCK-8) solution (CK04, Dojindo Laboratories, Kumamoto, Japan), and incubated for 1 h at 37 °C. Finally, 200 μL solution in each well was in order transferred into another 96-well plate, and the absorbance optical density (OD) value at 450 nm (OD_450nm_) was measured by a NanoDrop 2000/2000c spectrophotometer (Thermo Fisher Scientific, Waltham, MA, USA). Every assay was performed in triplicate.

### Cell vitality assay

The non-contact co-culture method was used for cell vitality assay and following experiments. The untransfected HUVECs (2×10^5^/well) were cultured in the lower chamber of a 24-well transwell plate with 3 μm pore insert and labeled with Did fluorescent probe. The remaining experimental operations were the same as TEM assay.

### DNA agarose gel electrophoresis assay

The co-cultured HUVECs in the lower chamber were washed, harvested, and centrifugated for 1 min at 10,000 rpm/min. The supernatant was collected and used for enzyme-linked immunosorbent assay (ELISA) and the remaining cells in the lower layer were used for electrophoresis assay. The genomic DNA of HUVECs was extracted by using the TIANamp Genomic DNA Kit (DP304, Tiangen Biotech Co., Ltd., Beijing, China) according to the manufacturer’s protocol. Finally, DNA (15 μl/lane) was examined by electrophoresis on a 1.5% agarose gel. The electrophoresis was performed in 1×TBE running buffer at a constant voltage of 100 V for 50 min. The image was obtained using a Tanon 1600R Gel Imaging System (Shanghai, China). Every assay was performed in triplicate.

### ELISA

The concentrations of SDC-1 and TNF-α in supernatant of co-cultured HUVECs were detected by ELISA according to the manufacturer’s protocol. The human SDC-1 (ml062743) and TNF-α ELISA Kit (ml077385) were purchased from Shanghai Enzyme-linked Biotechnology Co., Ltd. (Shanghai, China).

### Detection of apoptotic cells by flow cytometry

Apoptosis of HUVECs was evaluated by the Annexin V-APC/PI Apoptosis Detection Kit (22838, AAT Bioquest, Sunnyvale, CA, USA). The co-cultured HUVECs were harvested and stained with 10 μL Annexin V-APC & PI for 15 min in the dark according to the description provided by the manufacturer. The fluorescence-stained cell population and apoptotic index were determined by a Guava easyCyte™ 8HT flow cytometry (Merck Millipore Corporation, Billerica, MA, USA).

### Observation of cellular and subcellular morphology

The co-cultured HUVECs in lower chamber were harvested, and centrifugated for 8 min at 800 rpm/min. The supernatant was abandoned. The remaining HUVECs were divided into two parts, one of which was used for fluorescence observation. The other was fixed with stationary solution (2.5% glutaraldehyde in 0.2 M HEPSE) for 2 h at 4 °C, washed in 0.1 mol/L PBS for three times. The cells were then fixed in 1% osmic acid for 2 h, dehydrated with gradient alcohol and acetone, soaked in propylene oxide, embedded in SPI-Pon812 epoxy resin (90529-77-4, SPI-Chem, PA, USA), cut into 1-μm-thick sections using an UC7 ultramicrotome (Leica, Wetzlar, Germany) and stained with uranyl acetate-lead citrate. The cells were then observed using a transmission electron microscope (HT7700 120-kV, Hitachi Co., Ltd., Tokyo, Japan).

### Double immunofluorescent analysis

Double immunofluorescent analysis was performed to detect SDC-1 and TNF-α in co-cultured HUVECs. The cells were fixed with 4% formaldehyde for 30 min, permeabilized with 0.5% Triton X-100 in PBS for 15 min, washed 3 times in PBS and blocked with 10% normal donkey Serum/PBS for 30 min at room temperature (RT). The HUVECs were then stained with the rabbit-anti SDC-1 and mouse-anti TNF-α antibodies (10593-1-AP and 60291-1-AP, Proteintech Group, Inc. Rosemont, IL, USA) at a dilution of 1:100 for 1 h at RT, washed 3 times with PBS and then, incubated with their secondary antibodies prepared in 3% BSA in PBS at a dilution of 1:200 for 1 h at RT. Alexa Fluor Plus 555 donkey anti-rabbit IgG (SDC-1) was from Abcam (ab150074, Cambridge, MA, USA), and Alexa Fluor® 594 affinipure donkey anti-mouse IgG (TNF-α) from Jackson ImmunoResearch Laboratories, Inc. (715-585-150, West Grove, PA, USA). After washing, the nuclei were stained with 15 μL of 4′,6-diamidino-2-phenylindole (DAPI). The cells were then observed by a fluorescence microscope (Olympus Optical Co., Ltd., Tokyo, Japan). The images contain overlay of SDC-1 (yellow), TNF-α (red), and nuclei (blue). Six fields were randomly selected to count the number of DAPI-stained cells.

### Animal experiments

All animal experiments were approved by the Animal Ethics Committee of Yijishan Hospital and performed according to our previous method^3^. Twelve BALB/c-nu nude mice (females, aged 4 weeks) [SCXK (hu) 2017-0018, Lingchang Biotechnology, Shanghai, China] were intraperitoneally injected with empty vector-transfected HCCLM3 cells, and randomly divided into two groups (*n* = 6 in each group). One week later, the mice of two groups were intraperitoneally injected with equal amounts of normal saline (NS group) and necrostatin-1 (Nec-1, HY-15760, MedChemExpress, Monmouth Junction, NJ, USA) (Nec-1 group) (both 1 μg/g per day for 4 weeks), respectively. Six weeks after HCC cells were inoculated, the abdominal organs were obtained. H&E and CD31 IHC staining were carried out to evaluate the necrosis of liver MVECs. A scoring system for endothelial cell necrosis described in the literaturewas used^25^, in which 0 = within normal limits, 0.5 = minimal to slight, 1 = slight to mild, 2 = moderate, 3 = more or severe, and 4 = massive or very severe necrosis. Moreover, double immunofluorescent analysis of CD31 and MLKL was performed. Mouse anti-mouse CD31 antibody (ab24590) and rabbit anti-mouse MLKL (ab196436) was purchased from Abcam (Cambridge, MA, USA), FITC goat anti-mouse IgG (H + L) (AS001) and Cy3 goat anti-Rabbit IgG (H + L) (AS007) were from ABclonal (Boston, MA, USA). Ten size-fixed fields (0.1 × 0.1 mm^2^) were randomly selected to quantify the number of DAPI-stained cells.

### Statistical analysis

Results are presented as the means ± standard deviation. Statistical analyses were performed using SPSS 22.0 (SPSS, Chicago, IL, USA) and Graphpad prism 8.0 (GraphPad Software, La Jolla, CA, USA). Quantitative data were compared using Student’s *t* test or ANOVA. Categorical data were analyzed by chi-squared test and Fisher exact test. Kaplan–Meier curves were used to assess the recurrence or survival disparity between different subgroups. *P < 0.05, **P < 0.01, and ***P < 0.001 were considered statistically significant.

## Supplementary information

Supplementary table S1

Supplementary table S2

Supplementary Fig. S1

Legend of Supplementary Fig. S1
