# Supplementary material for: Heparanase induces necroptosis of microvascular endothelial cells to promote the metastasis of hepatocellular carcinoma
Source: Cell Death Discov. 2021 Feb 17;7:33. doi: 10.1038/s41420-021-00411-5 (PMC7889896; doi:10.1038/s41420-021-00411-5)
Supplement: Supplementary file 2 — Supplementary table S2 [file 41420_2021_411_MOESM2_ESM.docx]

**Table S2. Primers uesd in qRT-PCR**

| **Genetic  informations** | **Primer orientation** | **Primer sequence(5'-3')** |
| --- | --- | --- |
| [NM_001098540.2](https://www.ncbi.nlm.nih.gov/entrez/viewer.fcgi?db=nucleotide&id=315360643) | H-HPSE-S | TCCTCATCCTCCTGGGTTCTC |
|  | H-HPSE-A | AGGGCCATTCCAACCGTAACT |
| [NM_001101](http://www.ncbi.nlm.nih.gov/nuccore/168480144) | H-ACTIN-S | CACCCAGCACAATGAAGATCAAGAT |
|  | H-ACTIN-A | CCAGTTTTTAAATCCTGAGTCAAGC |
| [NM_001006946.1](https://www.ncbi.nlm.nih.gov/nuccore/NM_001006946.1) | H-SDC-1-S | ACGGCTATTCCCACGTCTC |
|  | H-SDC-1-A | TCTGGCAGGACTACAGCCTC |
| [NM_000594.4](https://www.ncbi.nlm.nih.gov/nuccore/NM_000594.4) | H-TNF-alpha-S | CCTGCCCCAATCCCTTTATT |
|  | H-TNF-alpha-A | CCCTAAGCCCCCAATTCTCT |
| [NM_002046.7](https://www.ncbi.nlm.nih.gov/nuccore/NM_002046.7) | H-GAPDH-S | TGACTTCAACAGCGACACCCA |
|  | H-GAPDH-A | CACCCTGTTGCTGTAGCCAAA |
| [NM_003804](https://www.ncbi.nlm.nih.gov/nuccore/1675002725) | H-RIPK1-S | TTACATGGAAAAGGCGTGATACA |
|  | H-RIPK1-A | AGGTCTGCGATCTTAATGTGGA |
| [NM_006871.4](https://www.ncbi.nlm.nih.gov/nuccore/1519241938) | H-RIPK3-S | AATTCGTGCTGCGCCTAGAAG |
|  | H-RIPK3-A | TCGTGCAGGTAAAACATCCCA |
| [NM_152649.4](https://www.ncbi.nlm.nih.gov/nuccore/NM_152649.4) | H-MLKL-S | AGGAGGCTAATGGGGAGATAGA |
|  | H-MLKL-A | TGGCTTGCTGTTAGAAACCTG |
| [NM_012311.3](https://www.ncbi.nlm.nih.gov/entrez/viewer.fcgi?db=nucleotide&id=359807087) | H-TNFR-S | TGCCTACCCCAGATTGAGAA |
|  | H-TNFR-A | ATTTCCCACAAACAATGGAGTAG |
| [NM_003789.4](https://www.ncbi.nlm.nih.gov/nuccore/NM_003789.4) | H-TRADD-S | GCTGTTTGAGTTGCATCCTAGC |
|  | H-TRADD-A | CCGCACTTCAGATTTCGCA |
| [NM_001372051.1](https://www.ncbi.nlm.nih.gov/nuccore/NM_001372051.1) | H-Caspase-8-S | CTCCCCAAACTTGCTTTATG |
|  | H-Caspase-8-A | AAGACCCCAGAGCATTGTTA |
| [NM_003824.4](https://www.ncbi.nlm.nih.gov/nuccore/NM_003824.4) | H-FADD-S | GCTGGCTCGTCAGCTCAAA |
|  | H-FADD-A | ACTGTTGCGTTCTCCTTCTCT |
| [NM_003998.4](https://www.ncbi.nlm.nih.gov/nuccore/NM_003998.4) | H-NF-κB-S | GAAGCACGAATGACAGAGGC |
|  | H-NF-κB-A | GCTTGGCGGATTAGCTCTTTT |
| [NM_002745.5](https://www.ncbi.nlm.nih.gov/nuccore/NM_002745.5) | H-p38MAPK-S | TCACACAGGGTTCCTGACAGA |
|  | H-p38MAPK-A | ATGCAGCCTACAGACCAAATATC |

**Abbreviations:**HPSE, heparanase; SDC-1, Syndecan-1; TNF-α, tumor necrosis factor-α; GAPDH, glyceraldehyde-3-phosphate dehydrogenase; RIPK, receptor-interacting protein kinase; MLKL, mixed lineage kinase domain-like protein; TNFR, tumor necrosis factor receptor; TRADD, TNFR associated death domain; FADD, Fas-associated death domain; NF-κB, nuclear factor kappa B; p38MAPK, p38 mitogen-activated protein kinase.
