# Supplementary figures and images for: Heparanase induces necroptosis of microvascular endothelial cells to promote the metastasis of hepatocellular carcinoma
Source: Cell Death Discov. 2021 Feb 17;7:33. doi: 10.1038/s41420-021-00411-5 (PMC7889896; doi:10.1038/s41420-021-00411-5)

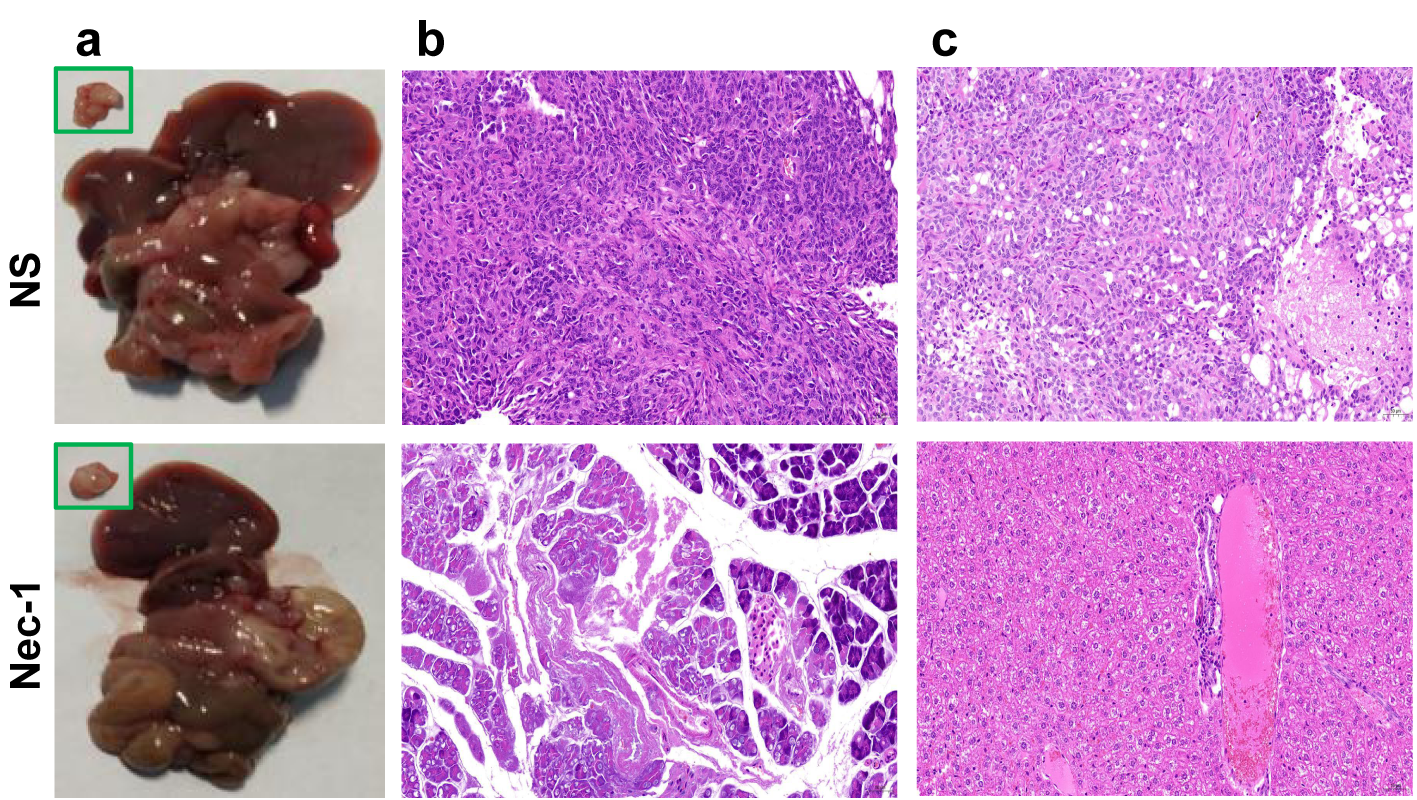

Supplement: Supplementary file 3 — Supplementary Fig. S1 [file 41420_2021_411_MOESM3_ESM.tif]
