# Supplementary material for: Heparanase induces necroptosis of microvascular endothelial cells to promote the metastasis of hepatocellular carcinoma
Source: Cell Death Discov. 2021 Feb 17;7:33. doi: 10.1038/s41420-021-00411-5 (PMC7889896; doi:10.1038/s41420-021-00411-5)
Supplement: Supplementary file 4 — Legend of Supplementary Fig. S1 [file 41420_2021_411_MOESM4_ESM.docx]

**Fig. S1. Tissue specimen of nude mice and HE staining. a** Tissue specimen from the abdominal cavity of nude mice of two groups. **b** HE staining of omentum (scale bars, 200 μm). HCC cells were found in two groups. **c** HE staining of liver tissues (scale bars, 200 μm). HCC cells were observed in NS groups, while no HCC cells were found in Nec-1 group.
